# Biological Nitrification Inhibitors with Antagonistic and Synergistic Effects on Growth of Ammonia Oxidisers and Soil Nitrification

**DOI:** 10.1007/s00248-024-02456-2

**Published:** 2024-11-20

**Authors:** Sulemana Issifu, Prashamsha Acharya, Jasmeet Kaur-Bhambra, Cecile Gubry-Rangin, Frank Rasche

**Affiliations:** 1 Institute of Agricultural Sciences in the Tropics (Hans-Ruthenberg-Institute), University of Hohenheim, Garbenstr. 13, 70599 Stuttgart, Germany; 2 School of Biological Sciences, University of Aberdeen, Cruickshank Building, Room 1.13, St Machar Drive, Aberdeen, AB24 3UU Scotland; 3 Present Address: International Institute of Tropical Agriculture (IITA), P.O. Box 30772-00100, Nairobi, Kenya; 4 Present Address: Department of Plant and Environmental Sciences, University of Copenhagen, Thorvaldsensvej 40, 1871 Frederiksberg C, Denmark

**Keywords:** Ammonia-oxidisers, BNI, Metabolite, Nitrification, Rhizosphere

## Abstract

Biological nitrification inhibition (BNI) refers to the plant-mediated process in which nitrification is inhibited through rhizospheric release of diverse metabolites. While it has been assumed that interactive effects of these metabolites shape rhizosphere processes, including BNI, there is scant evidence supporting this claim. Hence, it was a primary objective to assess the interactive effects of selected metabolites, including caffeic acid (CA), vanillic acid (VA), vanillin (VAN), syringic acid (SA), and phenylalanine (PHE), applied as single and combined compounds, against pure cultures of various ammonia-oxidising bacteria (AOB, *Nitrosomonas europaea*, *Nitrosospira multiformis*, *Nitrosospira tenuis*, *Nitrosospira briensis*) and archaea (AOA, *Nitrososphaera viennensis*), as well as soil nitrification. Additionally, benzoic acid (BA) was examined as a novel biological nitrification inhibitor. All metabolites, except SA, tested as single compounds, achieved varied levels of inhibition of microbial growth, with CA exhibiting the highest inhibitory potential. Similarly, all metabolites applied as single compounds, except PHE, inhibited soil nitrification by up to 62%, with BA being the most potent. Inhibition of tested nitrifying microbes was also observed when compounds were assessed in combination. The combinations VA + PH, VA + CA, and VA + VAN exhibited synergism against *N. tenuis* and *N. briensis*, while others showed antagonism against *N. europaea*, *N. multiformis*, and *N. viennensis*. Although all combinations suppressed soil nitrification, their interactions against soil nitrification revealed antagonism. Our findings indicate that both antagonism and synergism are possible in rhizospheric interactions involving BNI metabolites, resulting in growth inhibition of nitrifiers and suppression of soil nitrification.

**Supplementary Information:**

The online version contains supplementary material available at 10.1007/s00248-024-02456-2.

## Introduction

Nitrification, which is the oxidative conversion of reduced nitrogen (N) form (e.g., NH4+) to NO3– by nitrifying microorganisms [1–3], has been acknowledged for numerous environmental and ecosystem problems, including climate relevant nitrous oxide emissions [4], contamination of underground water, and eutrophication of surface water [5, 6]. Nitrification is most favoured under oxygen and moderate moisture (55–60% water-filled pore space) conditions [7].

Economically, nitrification compromises the efficient utilisation of fertilizer N by crops, leading to N losses that result in an annual economic loss of approximately US$15 billion in the USA [5] and £2.3 billion, approximating to half of annual agricultural profits in the UK [8]. Efforts to manage these problems have instigated the use of synthetic compounds to control nitrification and, thereby, reduce N losses from agricultural fields [5, 9, 10]. However, reliance on synthetic nitrification inhibitors has not been widely accepted due to economic reasons and questions of durability, sustainability, and safety [11–15]. Thus, a nature-based strategy to curtail nitrification is preferable [16].

There is ample evidence to suggest the existence of natural processes in the ecosystem that can suppress nitrification [17, 18]. Several plants, including but not limited to, grasses and trees, release bioactive metabolites into the soil that can inhibit nitrification [19, 20]. This process has been defined as biological nitrification inhibition (BNI) [21]. There are diverse mechanisms through which BNI metabolites can exhibit inhibitory action [9, 10, 22, 23] including, but not limited to, inhibition of the growth and activity of nitrifiers [24] and interference with the pathways of the nitrification process comprising ammonia monooxygenase (AMO) and/or hydroxylamine oxidoreductase (HAO) [20, 21].

BNI metabolites are very diverse in their chemical structure and include monoterpenes [20], phenolics [25], fusicoccane-type diterpenes [21, 26], amino acids [27], fatty alcohol [28], and other metabolites [17, 29, 30]. These discovered metabolites with verified BNI potential are also defined as “putative BNI metabolites” [23], some of them occurring widely in many organisms due to their diverse functional ecophysiological roles [9, 10, 31, 32]. Prominent putative BNI molecules include caffeic acid, ferulic acid, quercetin, isoquercitrin, *p*-coumarate, *p*-hydroxybenzoic acid [17, 23, 29, 30], *p*-benzochinone [14, 15], syringic acid [33], coumarin [34], vanillic acid [25], and vanillin [35, 36].

While most putative BNI metabolites have been studied individually [33], the discovery of the co-exudation of multiple metabolites, as observed in *Thinopyrum intermedium* [37] and *Brachiaria humidicola* [9, 10, 26], suggests a compelling assumption that co-exudated BNI metabolites, in particular, may amplify the BNI effect within crop rhizospheres [23]. In a recent study by Lu et al. [33], the simultaneous release of BNI metabolites syringic acid and 1,9-decanediol revealed a synergistic interaction in inhibiting the growth of *N. europaea* and *N. stercoris* as well as soil nitrification. While it is recognised that nitrifiers respond differently to different chemical and biological nitrification inhibitors [24, 38], understanding the extent of the impact of individual, in comparison to combined BNI metabolites on the growth of a variety of ammonia oxidisers, including AOA and soil nitrification remains elusive. Therefore, the aim of this study was to examine the divergent effects of multiple putative BNI metabolites secreted by *T. intermedium* [37], a perennial wheatgrass noted for its numerous ecosystem services [39–41] with an emphasis on their interactive influences on the growth of various strains of ammonia-oxidisers (AOB and AOA) and soil nitrification.

## Materials and Methods

### Selection and Preparation of Metabolites for Pure Culture Assays

Metabolites assayed in this study have been previously identified as BNI metabolites ([35, 23, 33]) and were found in coexistence at higher concentrations under NH_4_^+^ than NO_3_^–^ nutrition in the rhizosphere of hydroponically grown *T. intermedium* [37]. A combination of HPLC and GC–MS analyses were used for the detection and quantification of the metabolites. The phenolic compounds caffeic acid, vanillic acid, syringic acid, and vanillin, as well as the amino acid phenylalanine (Supplementary material 5, chemical structure of metabolites), which were all procured from Thermo Fisher Scientific (China), were tested for their inhibitory effects on pure cultures of nitrifiers, as single or in combined doses. Subsequent soil incubation tests were performed with selected metabolites (i.e. caffeic acid, vanillic acid, vanillin, phenylalanine in addition to benzoic acid) that revealed suppressive ability against AOB and AOA strains in the pure culture assays. Single metabolites were dissolved separately for the single metabolite effects assays. For interactive effect assays, two metabolites were combined pairwise at a 1:1 (w/w) ratio of 200:200 µM. Stock solutions of the metabolites (separate or combined) were prepared by dissolving pure substrates in 100% DMSO. Subsequently, the dissolved metabolites were used for microbial growth inhibition assays using multiple concentrations ranging between 100 and 1000 µM, depending on the strains (AOB and AOA). All metabolites were selected because of their concurrent release in the root exudates of *T. intermedium* [37], thereby justifying the need to understand the effect of their interaction.

### Microbial Growth Inhibition Assays

The procedure followed that of Kaur-Bhambra et al. [24] with a few modifications. Briefly, microbial strains tested included AOB strains *Nitrosomonas europaea* ATCC 19718, *Nitrosospira multiformis* ATCC 25196, *Nitrosospira tenuis* NV12 [42], *Nitrosospira briensis* 128 [43], and the AOA strain *Nitrososphaera viennensis* [44]. Modified freshwater media [45], with phenol red as pH indicator and Na_2_CO_3_ used for pH adjustments, was used for cultivating the AOB. Freshwater media [44] was used to grow *N. viennensis*. The nitrifiers were cultured in 30-mL universal plastic bottles using an inoculum of 1% in 10-mL liquid media. The cultures were supplemented with 10 µL from the corresponding stock to achieve the desired testable concentrations (200 µM and 200:200 µM) of metabolites and an acceptable concentration of DMSO (0.1% (v/v)). Subsequently, cultures supplemented with the metabolites were incubated at 28 °C and 35 °C for AOB and AOA, respectively. The AOA (*N. viennensis*) culture was given a lower concentration (four times less compared with AOB) of the metabolites due to the generally high sensitivity of AOA to BNI compounds [24]. Controls with similar conditions, except for the addition of BNI metabolites, were set up alongside. It was noticed that most of the metabolites altered the pH of the media of the AOB after introduction. When necessary, pH was adjusted with 10% (v/v) HCl and 5% (v/v) Na_2_CO_3_ to ensure similar starting pH (AOB =  ~ 7.4 and AOA =  ~ 6.5) conditions for all treatments (Supplementary material 1). The assays were set up in quadruplicates.

Microbial growth was recorded twice a day for AOB for 1 week, and once a day for AOA for 2 weeks as colorimetric quantification of nitrite using the Griess test [46]. Using a minimum of four timepoints within the log phase, maximum growth rate (µ_max_) was determined as a slope of log-linear plots of nitrite concentration versus time. Growth inhibition is expressed as follows:

$$\mathrm{Microbial}\;\mathrm{growth}\;\mathrm{inhibition}\;\left(\%\right)=\left(\frac{\mu avg.{Control}_{DMSO}-\mu avg.{Treatment}_{BNI}}{\mu avg.{Control}_{DMSO}}\right)\times100$$

### Soil Nitrification Experiments

Single metabolites and their pairwise combinations (except benzoic acid) of caffeic acid, vanillic acid, vanillin, and phenylalanine were tested in the soil for their effect on nitrification. For the soil nitrification experiment, agricultural soil was collected from Meiereihof, an experimental site of the University of Hohenheim (Stuttgart, Germany). It was a Luvisol (silt loam texture (3.4% sand, 76.2% silt, 20.5% clay), with the following chemical characteristics: pH 6.8; total carbon (C) and N of 1.11% and 0.09%, respectively, C/N ratio of 12; organic C of 1.03%. The soil was air-dried and passed through a 2-mm sieve before use. Similar to previous studies [27, 35, 36, 47], 10 g of dry soil was weighed into a 10 ml glass vials and supplied with the metabolites (Sigma Aldrich, Darmstadt, Germany) at concentrations of 500 μg g^−1^ soil. For the interactive effect assessments, two metabolites were added to the dry soil contemporaneously at a ratio of 1:1 w/w before rewetting. The mixtures were shaken vigorously to ensure homogeneity of the contents, and supplied with dissolved NH_4_(SO4)_2_ at a concentration of 182 mg N kg^–1^. The vials were sealed with a parafilm perforated with a pin on the top to allow aeration during the experimental period. A control treatment without any metabolite was established. Each vial was provided with 1 mL of deionized water initially after amending with the metabolites and then 200 μL of distilled water every 9 days to achieve and maintain a 60% water-filled pore space (WFPS) [48]. All treatments were incubated at 28 °C in the dark for 22 days. NH_4_^+^ and NO_3_^–^ were quantified colorimetrically following a previous protocol [35, 36]. Experiments were set up in triplicates. Relative nitrification inhibition (RNI) with modifications [49] to assess the comparative effects of the combined and single metabolites against nitrification was calculated using four timepoints from day 4 (second timepoint) (Supplementary material 2) as follows:

$$\mathrm{RNI}\;(\%)=\left(\frac{\mu avg.Control-\mu avg.{Treatment}_{BNI}}{\mu avg.Control}\right)\times100$$

### Analysis of the Interactive Effects of Metabolites on Microbial Growth and Soil Nitrification

Procedures were generally adopted from Lu et al. [33] with some modifications. Preparations of metabolites for supplementation have been described above. Interactive effect analyses were performed using the effect-based approach published by Foucquier and Guedj [50]. The mathematical model, known as the Jin equation, was adopted from Wu et al. [51] and is expressed as follows:

$$q={\mathrm{E}}_{\mathrm{A}+\mathrm{B}+...}/\left({\mathrm{E}}_{\mathrm{A}}+{\mathrm{E}}_{\mathrm{B}}+... - {\mathrm{E}}_{\mathrm{A}}\times {\mathrm{E}}_{\mathrm{B}}\times ...\right)$$

where E_A+B+…_, E_A_, E_B_,_…_ are the average effects of the combination treatments and individual treatments of the selected metabolites A and B, respectively. According to Wu et al. [51], the *q* values < 0.85, 0.85–1.15, and ≥ 1.15 represent antagonism, additivity, and synergism, respectively.

### Statistics

Statistical analyses were done with JMP Pro 17 software, while GraphPad Prism 10.0.2 (232) was used for visualisation. All data were subjected to outlier checks within treatment replicates. Subsequently, the homoscedasticity of all data was checked by a graphical screening of the residuals and Q-Q plots to ensure the assumption of normality is satisfied. ANOVA was conducted, followed by a post hoc Tukey test to estimate the statistical significance of the effects of single and combined metabolites on microbial growth, relative nitrification inhibition, NO_3_^–^ accumulation, and NH_4_^+^ concentration. Results are presented as least square means ± standard error of means (LSM ± SE).

## Results

### Effects of Single Metabolites on Nitrifier Growth

**Fig. 1:**
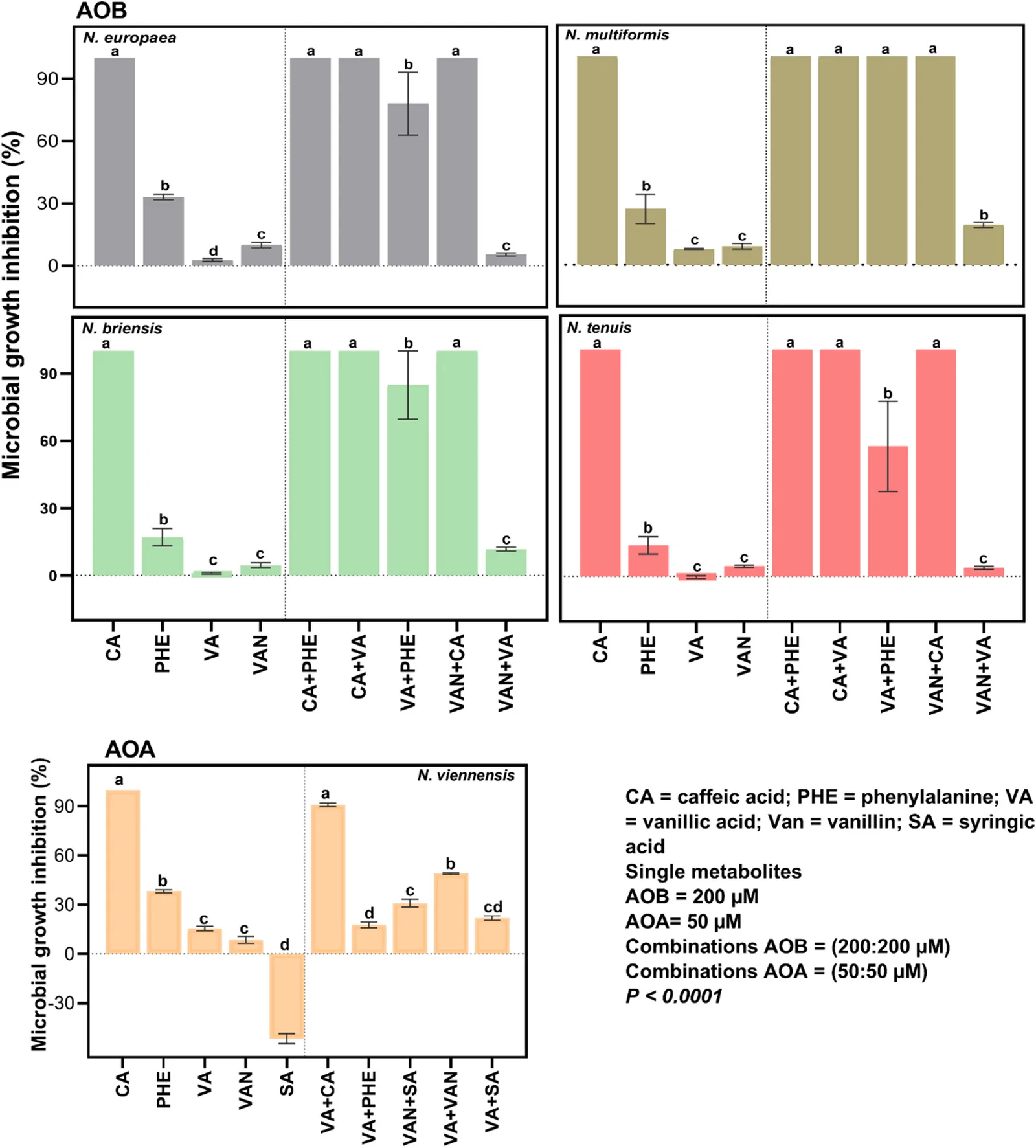
Single and combinatorial effects of metabolites on nitrifiers. Results are presented as least square means (± S.E.) of four replicates for each strain separately. Metabolite concentrations tested for AOA and AOB were 50 and 200 µM, respectively. Levels not connected by same letters within each strain are significantly different

Caffeic acid (CA), phenylalanine (PHE), vanillic acid (VA), vanillin (VAN), and syringic acid (SA) were tested as single compounds for their inhibitory effects on the growth of AOB (200 µM) and AOA (50 µM) (Supplementary materials 3). Except for SA, which showed stimulatory to no effect (− 0.63–6% for AOB and − 51% for AOA), all metabolites exhibited varying degrees of growth inhibition (*p* < 0.0001) (Fig. 1). CA showed the highest and most consistent inhibitory effect at 100% inhibition. CA had a lower inhibitory (2–52%) effect on AOB at concentrations of 50 µM (Supplementary Material 3). Comparatively, PHE inhibited all strains, with inhibition between 14 and 38%. Similarly, VAN and VA at a concentration of 200 µM achieved up to 10% and 8% inhibition of the AOB strains, while at 50 µM, VAN and VA inhibited *N*. *viennensis* up to 9% and 15%, respectively. However, VA showed inconsistency on the strains because of its minor effect (− 0.4 and − 1%) on *N. tenuis*, whereas VAN achieved consistent growth inhibition (4–10%) across all test strains. When tested at higher concentrations (1000 µM), all metabolites, except SA, achieved inhibitions up to 100% (*p* < 0.0001) (Supplementary Material 3) of the AOB. SA had the weakest effect as it did not inhibit any AOB strain under the comparative test conditions. But intriguingly, when tested at 80 and 160 µM, SA achieved an average inhibition of up to 16% against *N. viennensis* (Supplementary Material 3) even though at 50 µM, it failed to inhibit the AOA (− 51%). Overall, nitrifiers responded differently to the exposure to the tested metabolites.

### Interactive and Inhibitory Effects of Combined Metabolites

**Fig. 2:**
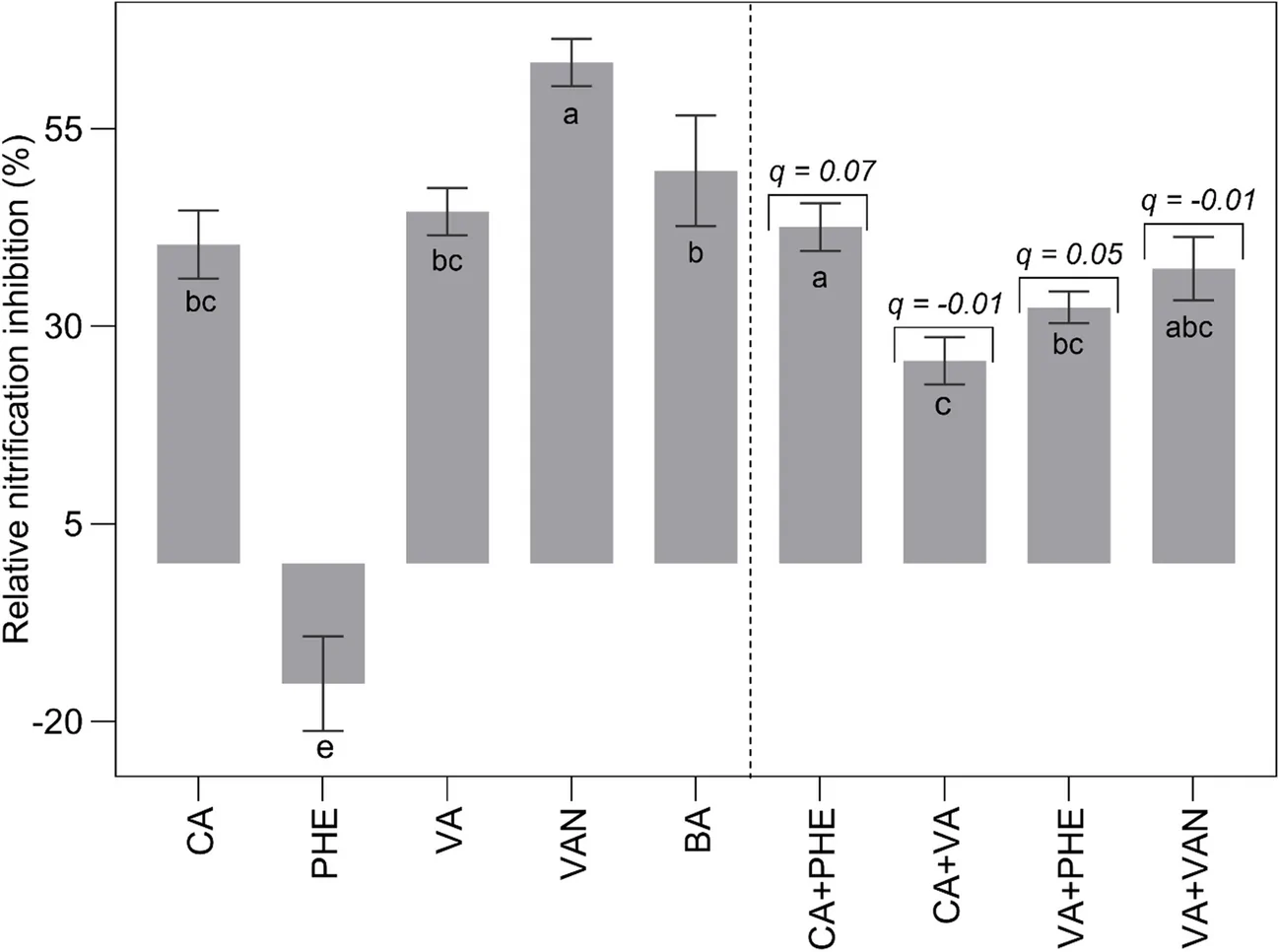
Percentage inhibition and interactive effects of metabolites on soil nitrification. Notes: *CA* caffeic acid, *PHE* phenylalanine, *VA* vanillic acid, and *VAN* vanillin. The operative sign + is used to signify combinations of the metabolites. Results are presented as least square means (± S.E.) of three replicates. Significance is determined for NO_3_^–^-N at *p* < *0.05*. Interpretation of *q* values (Jin equation); < 0.85 means antagonistic interaction; 0.85–1.15 means additive interaction; ≥ 1.15 means synergistic interaction

Almost all pairwise combinations resulted in inhibitions (Fig. 2), mostly interacting antagonistically (Table 1, Supplementary Material 4). Compared with the single use of CA, CA + VA, CA + VAN, and CA + PHE combinations achieved similar outcomes as sole CA, without any significant differences for all nitrifiers. Combinations with CA achieved the highest (100%) (*p* < 0.0001) inhibitions compared with all the other combinations (Supplementary Material 4). VA + PHE achieved higher (*p* < 0.0001) inhibitions of all AOB (up to 100%) than the separate use of each metabolite (VA = 9%, PHE = 41%). Conversely, for AOA, single metabolites (VA = 15%, PHE = 38%) achieved higher inhibitions (*p* < 0.0001) than the combined one (VA + PHE = 13%). The combined metabolites (VA + VAN) showed varying effects on the strains compared to the single metabolites (VA, VAN). This treatment was the least effective against the nitrifiers compared to all other combinations. However, it achieved an averagely higher inhibitions (16%) than the average inhibitions of the single metabolites (VAN = 7%, VA = 7%). In the AOA, VA + VAN achieved a higher effect (49%) (*p* < 0.0001) than the metabolites used individually, while in the AOB, no significant differences were observed (Supplementary material 4). SA with either VAN or VA (SA + VAN, SA + VA), only tested on AOA, achieved inhibitions of 31% and 22%, respectively, even though SA interestingly did not achieve inhibitions as a single metabolite at tested concentration of 50 µM. The effect of SA + VAN on AOA was significant (*p* < 0.0001) compared with the effects of the single metabolites, while the effect of SA + VA was only higher than SA (*p* < 0.0001), but not VA. Also, VA + PHE achieved 18% inhibition of *N. viennensis*.

**Table 1:** Interactive effects of metabolite combinations on nitrifiers

Combinations	*N. europaea*	*N. multiformis*	*N. briensis*	*N. tenuis*	*N. viennensis*
CA + PHE	− 0.03	− 0.04	− 0.06	-0.08	− 0.06
CA + VAN	− 0.11	− 0.13	− 0.29	− 0.30	− 0.29
CA + VA	− 0.57	− 0.04	14.00	0.72	− 0.06
VA + VAN	− 0.36	− 0.38	9.60	0.64	− 0.45
VA + PHE	− 1.38	− 0.59	42.54	3.12	− 0.03
VA + SA					0.03
VAN + SA					0.08

*CA* caffeic acid, *PHE* phenylalanine, *VA* vanillic acid, *VAN* vanillin, and *SA* syringic acid. The operative sign + is used to signify combinations of the metabolites. Interpretation of *q* values (Jin equation); < 0.85 means antagonistic interaction; 0.85–1.15 means additive interaction; ≥ 1.15 means synergistic interaction [51]

Interactively, combinations of most metabolites with CA (CA + PHE, CA + VAN, CA + VA) exhibited antagonistic interactions (*q* =  − 0.03–0.72) on the growth of all nitrifiers (Table 1). An exception was observed for *N. briensis,* revealing synergism (*q* = 14) of CA + VA. The combination of VA + PHE exhibited antagonistic interactions (*q* =  − 0.2 to − 1.38) on all nitrifiers. An exception was *N. briensis* (*q* = 42.54) and *N. tenuis* (*q* = 3.12), for which synergism was observed. Interaction between VA and VAN (VA + VAN) was antagonistic (*q* =  − 0.27–0.64) for all nitrifiers, except for *N. briensis* revealing a synergistic effect (*q* = 9.60). Combinations of SA with either VAN or VA (SA + VAN, SA + VA), tested only on *N. viennensis*, resulted in antagonistic interactions (VAN + SA, *q* = 0.08, SA + VA, *q* = 0.03).

### Soil Nitrification

Nitrification amended with metabolites was reduced in most soils in comparison to the untreated control for all timepoints, with a slower accumulation of NO_3_^–^ and a concomitant reduction in NH_4_^+^ concentration (Supplementary Material 5) (*p* < *0.0001*). An exception was the soil treated with PHE, revealing a negative effect on nitrification (− 15%) (Fig. 2) (*p* < 0.0001). VAN-amended soil showed reduced nitrification by 63%, followed by BA (50%), VA (44%), and CA (40%) (*p* < *0.0001*). Among the tested metabolite combinations, PHE + CA, VA + CA, VAN + VA, and PHE + VA achieved 43%, 26%, 37%, and 32% reduced nitrification, respectively (*p* < 0.0001). Accordingly, all combinations resulted in antagonistic interactions (PHE + CA, *q* = 0.07; VA + CA, *q* =  − 0.01; VAN + VA, *q* =  − 0.01; PHE + VA, *q* = 0.05) (*p* < 0.0001). Overall, soils amended with single metabolites (BA, VAN) achieved the highest reduction in nitrification than all combined metabolites (*p* < *0.0001*).

## Discussion

### Inhibitory Potential of Single Metabolites Against Nitrifiers

The presented study revealed the inhibitory potential of a range of BNI metabolites in pure cultures of both ammonia-oxidising bacteria (AOB) and archaea (AOA) as well as in soil. Among the metabolites tested, caffeic acid (CA), a hydrophilic metabolite, revealed the strongest effect on the growth of tested nitrifier strains and soil nitrification, a finding in line with Kolovou et al. [38], as well as Rice and Pancholy [30]. CA is a widely known bioactive metabolite occurring in nature [52], with allelopathic properties against various microbes, including but not limited to *Escherichia coli*, *Staphylococcus aureus*, and *Bacillus subtilis* [53, 54], and plants [55, 56]. The actual mode of action of CA remains elusive.

It is proposed that CA may inhibit ammonia oxidisers by acting as a cell permeabilizer, as reported in *E. coli* and *S. aureus* [53]. Sueishi et al. [57] demonstrated that CA is linked to nitric oxide scavenging, suggesting it as a mechanism for inhibiting nitrification in soil. As NO is an obligate intermediate in the oxidation of hydroxylamine (NH_2_OH) by hydroxylamine oxidoreductase (HAO) in AOB [58], it could be suggested that CA suppresses the HAO pathway of the nitrification process. This is further supported by the discovery that CA, even at a much lower concentration, differentially inhibited the oxidation of ammonia by both AOB and AOA [59]. The mechanism underlying the scavenging property of CA against NO during nitrification has not been well-investigated. However, it is assumed that due to its antioxidant property, which makes it reactive to reactive nitrogen species (RNS) like NO, CA reacts with NO to make it less oxidative [60].

Vanillin (VAN) and vanillic acid (VA) showed similar inhibitory properties against microbial growth in the pure cultures, as well as suppression of NO_3_^–^ production [25, 35, 36]. Here, we have provided further evidence of the inhibitory properties of VAN by ascertaining its impact on multiple ammonia-oxidising strains, thereby extending its known antimicrobial ability against multiple microbes [35, 36, 61, 62]. Depending on the species, VAN inhibits microbes such as *E. coli, Lactobacillus plantarum*, and *Listeria innocua* by altering ion gradients of microbial membranes and suppressing respiration [63]. Interestingly, VAN, relative to CA and phenylalanine (PHE), did not exert a strong inhibitory effect in the pure cultures, but it revealed the strongest nitrification inhibition in soil. This observed behaviour of VAN is directly opposite to our findings relating to PHE. VAN, being a phenolic metabolite of similar biosynthetic pathway as VA [64, 65], may share a similar soil nitrification inhibitory mechanism, such as the blocking of ammonia monooxygenase (AMO) pathway as reported in SA [33]. Another possibility includes stimulating other microbes [66] to outcompete nitrifiers, thereby reducing nitrification [35, 36]. PHE exhibited a significant suppression of the growth of all tested AOA and AOB strains, but did not suppress nitrification activity in the soil. PHE has been described for its antibacterial properties [67, 68], through cytotoxicity and potential ability to depolarise the microbial membrane [67]. While PHE has been characterised as a biological nitrification inhibitor [23], soil nitrification was not consistently inhibited [27]. This fact raises concern about its classification as a nitrification inhibitor. Comparatively, our findings on PHE are similar to what has been reported on sakuranetin in *Sorghum bicolor*, which was found to be effective in vitro against pure cultures but not in the soil [14, 15].

It is important to note that SA, which showed a weak inhibitory potential against AOB under the test conditions (pH ~ 7.4) and AOA when tested at 50 µM, exhibited an ability to inhibit AOA when tested at a higher concentration. The observed effect against AOA at a higher concentration supports the reported effectiveness of SA under acidic conditions [33] since AOA media (pH ~ 6.5) is comparatively acidic relative to AOB media (pH ~ 7.5–8). AOB and AOA are known to occupy different ecological niches [2], and are affected differently by environmental conditions, such as pH, which has been attributed to differences in the physiology of the two microbial groups [2, 69]. The foregoing brings into focus the issue about the role of the type and physicochemical properties of soil in the bioactivity of nitrification inhibitors against nitrifiers and nitrification. For instance, it has been reported that under brown, red, and cinnamon soils, nitrification inhibitors achieved different inhibitory effects—with pH and soil organic matter playing crucial roles [70]. It may be thus inferred that, under different soil conditions, BNI metabolites may act differently. Relatedly, the varying effects of SA, VAN, PHE, and VA on different AOB and AOA strains, as well as soil nitrification, underscore the need to re-evaluate BNI studies taking into account the differential microbial responses and their complexities. Benzoic acid (BA) suppressed NO_3_^–^ accumulation in the soil incubation study. Our study provides evidence of its ability to inhibit nitrification in NH_4_^+^-supplied soils. This suggests that BA may effectively suppress AOB, microbes primarily thriving under NH_4_^+^ conditions [27, 71]. Given that BA was identified as one of the metabolites released in the rhizosphere of *Thinopyrum intermedium* [37], there is a need for future research to understand its mode of action and its inhibitory potential against nitrifier growth. Overall, our findings indicate that not all metabolites showing strong antimicrobial activity in pure cultures may inhibit soil nitrification and vice versa.

### Interactive Effects of Metabolites in Pure Cultures

It was generally believed that an interactive effect on nitrifying microbes and nitrification arising from the coexistence of biochemically distinct BNI metabolites exists in the rhizosphere [23]. In our study, metabolite combinations largely resulted in antagonistic interactions. However, CA + VA, VA + VAN, and VA + PHE exhibited synergism against *N. briensis* and *N. tenuis*, respectively. This contrasting effect of the combinations on tested microbes resembles findings from other combinatorial studies, where combinations yielded synergistic and additive effects, respectively on *E. coli* and *Bacillus cereus* [72]. Further, metabolite combinations have resulted in contrasting effects, i.e. an additive effect against *Pseudomonas putida* and an antagonistic effect against *B. cereus *([72, 73]). This may be due to the differences in sensitivities to metabolites by different nitrifying microbes [24, 38]. The difference in sensitivity has been postulated to be associated with the differences in the chemical structures of the metabolites [24], as observed in AOA and AOB for different synthetic nitrification inhibitors [74]. There is currently no evidence to support the role of chemical structure. We suggest that the variation in microbial sensitivity could also be due to differences in their cellular structures. This suggestion is premised on earlier reports that have associated differences in AOB and AOA responses to environment conditions to their physiological structures [2, 69, 71]. In particular, VA and VAN combinations with other antimicrobial substances (e.g. gentamicin, spectinomycin) have been reported to synergistically inhibit the microbial growth of Gram-positive and Gram-negative bacteria [62]. This aligns with our finding that the combination of the two compounds resulted in synergism against the Gram-negative *N. briensis* and *N. tenuis*.

Noteworthy, the combinations with CA mostly resulted in total inhibitions of the test strains. This highlights the potency of CA as a domineering metabolite compared to other metabolites. Antagonism is mostly caused by a single metabolite dominating the others in a combination, a behaviour known as single agent dominance (SAD) [75]. Combinations used in our study did not result in synergism on soil nitrification, though they reduced nitrification. This partly confirms the findings in the pure culture assays and is contrary to the findings reported between SA and 1,9-decanediol, which resulted in synergism [33]. Thus, qPCR to estimate the of AOB and AOA abundances in the soil incubation studies along with RT-qPCR to evaluate respective gene expressions could provide additional insights about the interactive effects of the metabolites on the nitrifiers. From this, it could be concluded that not all BNI metabolites interact synergistically in the soil, which may be attributable to the single metabolite dominance effect [75]. Generally, antagonism between bioactive metabolites is not to be construed as a lack of effect, while synergism should not be construed as evidence of effect [75]. Notwithstanding, the chosen methodological approach, including the doses utilised in the combinations, could explain the observed antagonistic or synergistic outcomes.

## Conclusion and Outlook

Our study unveiled the potential for both antagonistic and synergistic interactions among BNI-inducing metabolites co-exuded by BNI-positive plants against nitrifying microbes. The approach used and the findings in this study contribute to the ongoing efforts to address the complexities involved in BNI investigations. Further, based on our findings, it could be inferred that antagonistic interactions between nitrification inhibitors do not compromise their combined inhibitory potential against nitrifiers and nitrification. This comprehension offers valuable insights for future breeding strategies to enhance BNI-positive crops specifically focusing on target metabolites. Prospectively, ongoing breeding endeavours should encompass a broad spectrum of metabolites, considering their interactions rather than solely concentrating on individual or novel metabolites. Investigations are needed to elucidate the mode of action of the metabolites utilised in this study in addition to other co-exuded nitrification inhibitors, whether singularly or in combination, ultimately benefiting breeding programs. Finally, it is important to investigate the physiological and ecological basis for the differential responses of nitrifying microbes (AOB and AOA) to BNI metabolites in order to harness the full potential of nitrification inhibitors.

## Supplementary Information

Below is the link to the electronic supplementary material.

Supplementary material 1: pH adjustments by metabolites (XLSX 11 kb)

Supplementary material 2: Calculations of %Relative Nitrification Inhibition (XLSX 32 kb)

Supplementary material 3: Growth curves and inhibition calculations (XLSX 456 kb)

Supplementary material 4: Comparisons single, combined metabolites, and q values calculations (XLSX 19 kb)

Supplementary material 5: NH4-NO3 dynamics over time (PDF 153 kb)

## Data Availability

No datasets were generated or analysed during the current study.
