# Supplementary material for: Biological Nitrification Inhibitors with Antagonistic and Synergistic Effects on Growth of Ammonia Oxidisers and Soil Nitrification
Source: Microb Ecol. 2024 Nov 20;87:143. doi: 10.1007/s00248-024-02456-2 (PMC11579066; doi:10.1007/s00248-024-02456-2)

Biological Nitrification Inhibitors with Antagonistic and Synergistic Effects on Growth of Ammonia Oxidisers and Soil Nitrification

Journal: Microbial Ecology

Sulemana Issifu[1], Prashamsha Acharya[1], Jasmeet Kaur-Bhambra[2], Cecile Gubry-Rangin[2], Frank Rasche[1,3]

[1]Institute of Agricultural Sciences in the Tropics (Hans-Ruthenberg-Institute), University of Hohenheim, Garbenstr. 13, 70599 Stuttgart, Germany.

[2]School of Biological Sciences, Cruickshank Building, Room 1.13, University of Aberdeen, St Machar Drive, Aberdeen, AB24 3UU, Scotland.

[3]International Institute of Tropical Agriculture (IITA), P.O. Box 30772-00100, Nairobi, Kenya.

Corresponding authors: Frank Rasche (f.rasche@cgiar.org) and Cecile Gubry-Rangin (c.rangin@abdn.ac.uk)

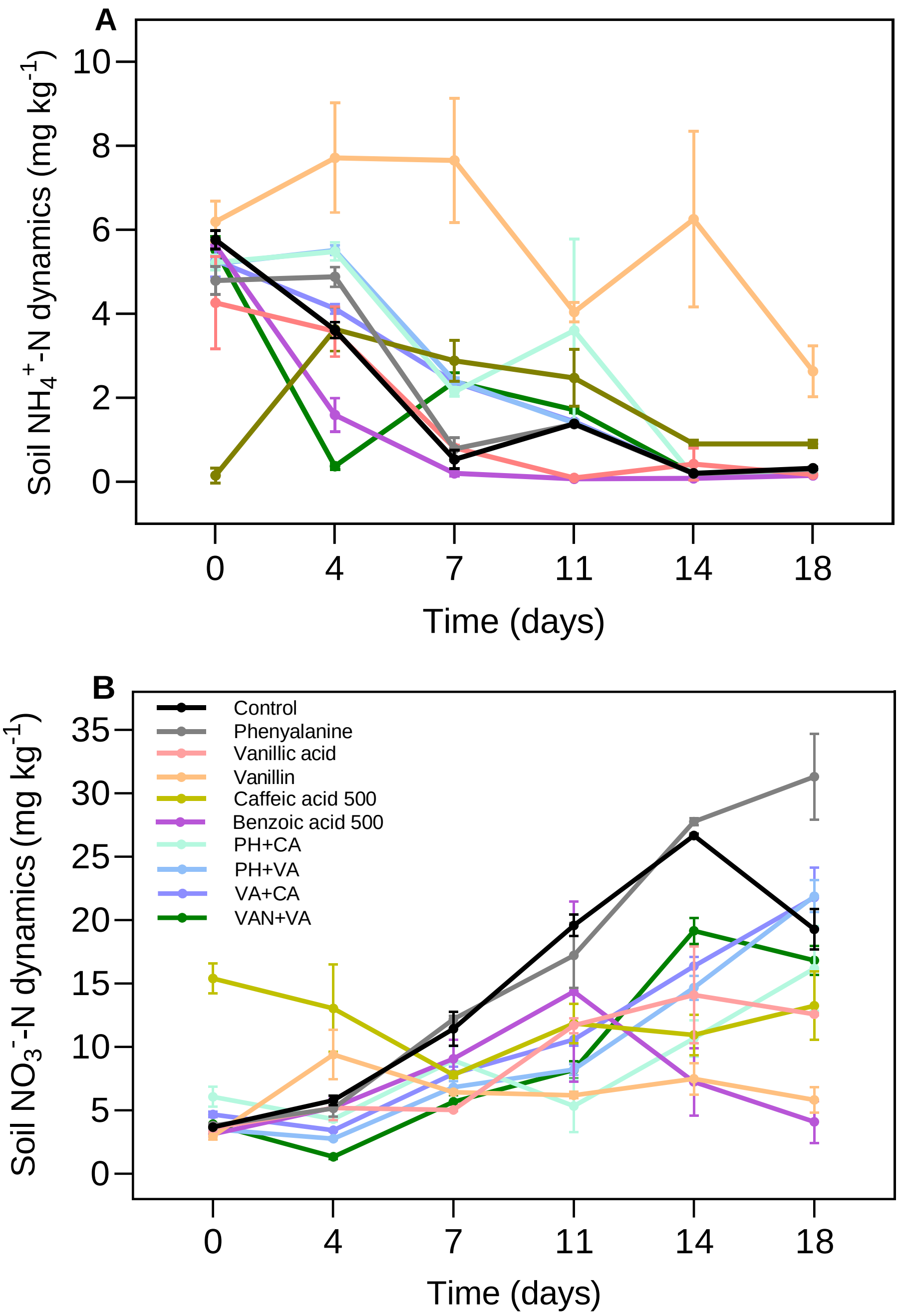

Fig. Dynamics of (A) NH4+ and (B) NO3 - in soils amended with metabolites. Notes: CA = caffeic acid, PHE = phenylalanine, VA = vanillic acid, VAN = vanillin, and SA = syringic acid. The operative sign + is used to signify combinations of the metabolites. Bars represent standard errors.

Structures of tested molecules in the study

Vanillic acid

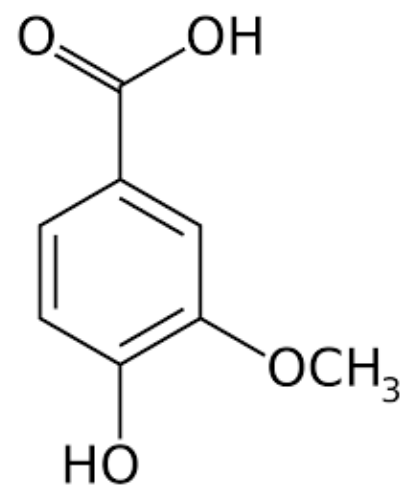

Caffeic acid

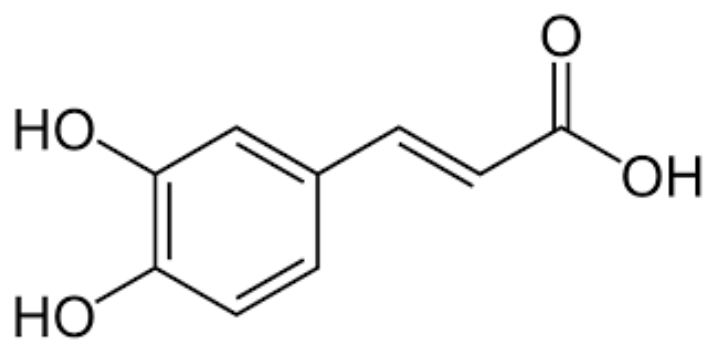

Phenylalanine

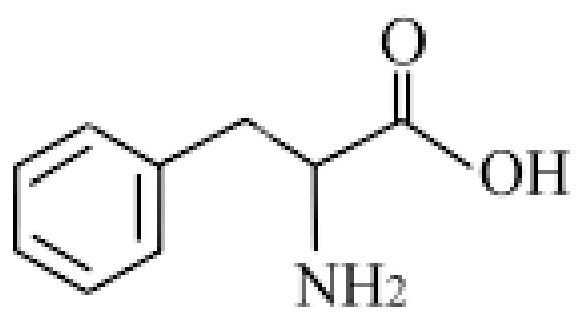

Benzoic acid

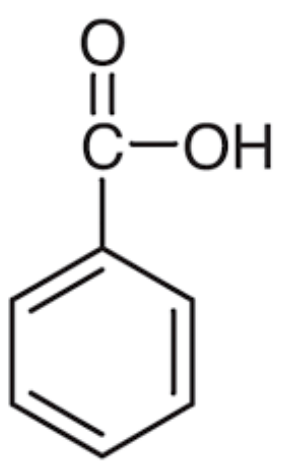

Vanillin

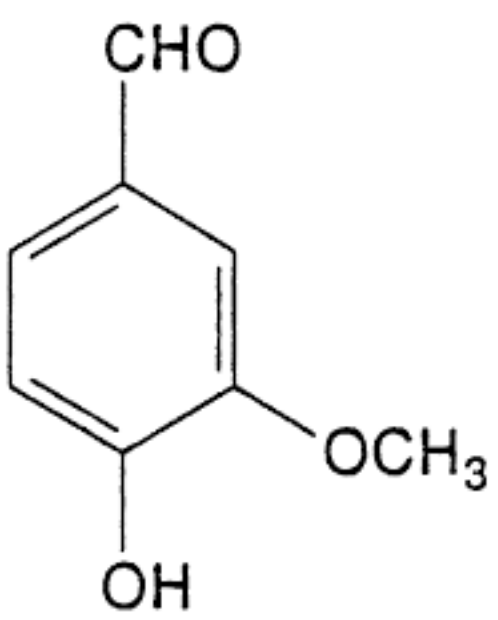

Supplement: Supplementary file 1 [file 248_2024_2456_MOESM5_ESM.pdf]
