# Supplementary material for: Biological Nitrification Inhibitors with Antagonistic and Synergistic Effects on Growth of Ammonia Oxidisers and Soil Nitrification
Source: Microb Ecol. 2024 Nov 20;87(1):143. doi: 10.1007/s00248-024-02456-2 (PMC11579066; doi:10.1007/s00248-024-02456-2)

**Biological Nitrification Inhibitors with Antagonistic and Synergistic Effects on Growth of Ammonia Oxidisers and Soil Nitrification**

**Journal: Microbial Ecology**

Sulemana Issifu[1], Prashamsha Acharya[1], Jasmeet Kaur-Bhambra[2], Cecile Gubry-Rangin[2], Frank Rasche[1,3]

[1]Institute of Agricultural Sciences in the Tropics (Hans-Ruthenberg-Institute), University of Hohenheim, Garbenstr. 13, 70599 Stuttgart, Germany.

[2]School of Biological Sciences, Cruickshank Building, Room 1.13, University of Aberdeen, St Machar Drive, Aberdeen, AB24 3UU, Scotland.

[3]International Institute of Tropical Agriculture (IITA), P.O. Box 30772-00100, Nairobi, Kenya.

**Corresponding authors:** Frank Rasche (f.rasche@cgiar.org) and Cecile Gubry-Rangin (c.rangin@abdn.ac.uk)

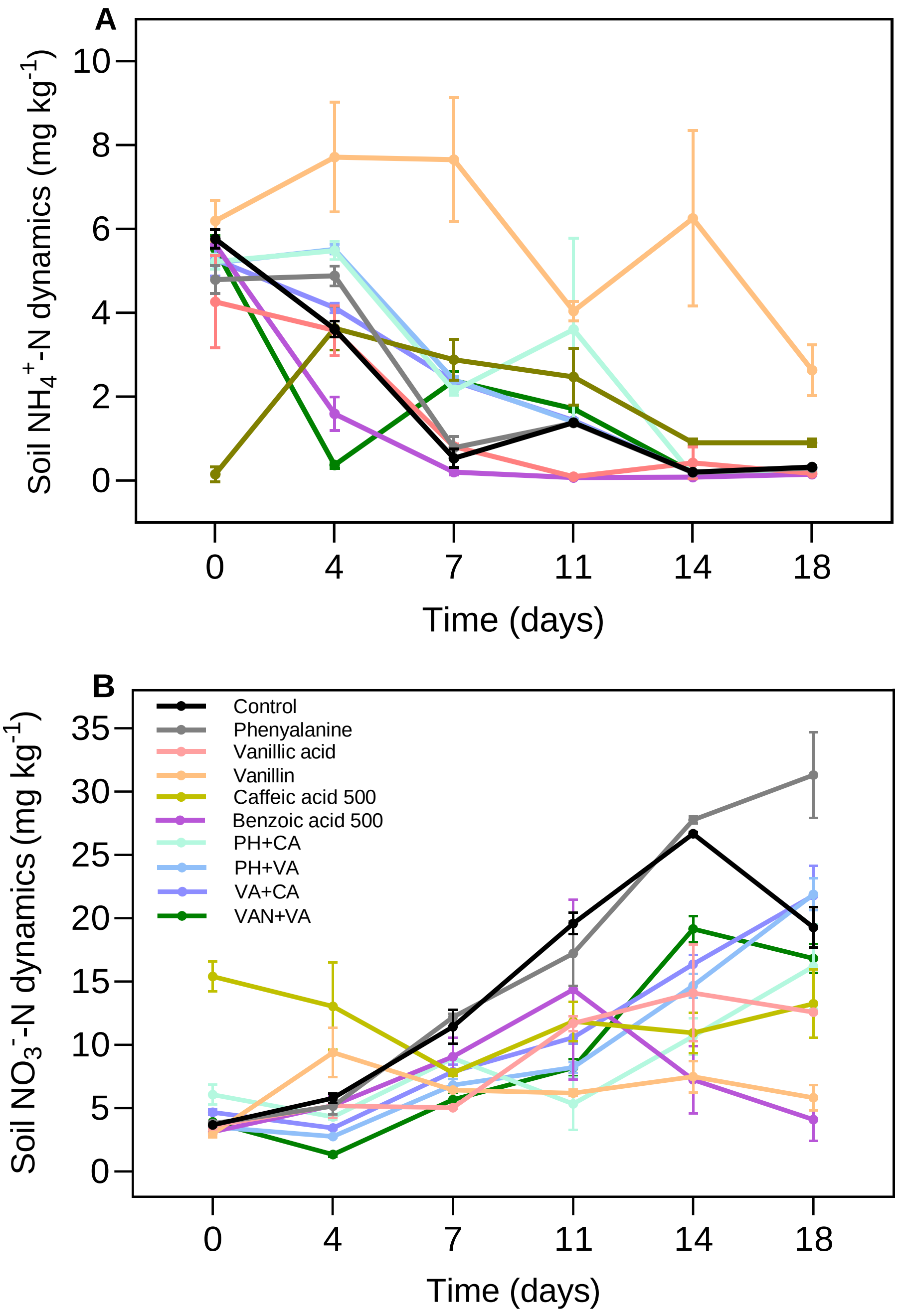


Fig. Dynamics of (A) NH4+ and (B) NO3 - in soils amended with metabolites. Notes: CA = caffeic acid, PHE = phenylalanine, VA = vanillic acid, VAN = vanillin, and SA = syringic acid. The operative sign + is used to signify combinations of the metabolites. Bars represent standard errors.

**Structures of tested molecules in the study**

Vanillic acid

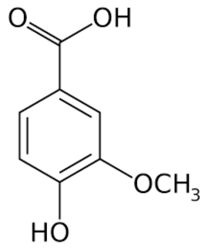


Caffeic acid

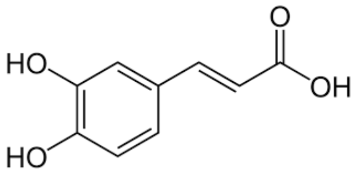


Phenylalanine

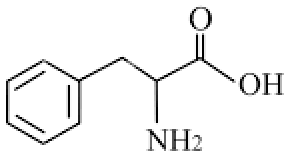


Benzoic acid

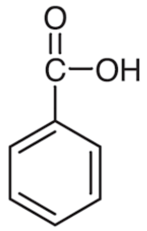


Vanillin

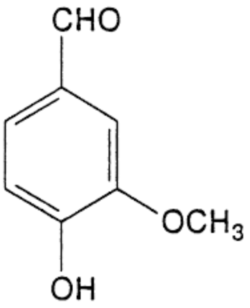

Supplement: Supplementary file 5 — Supplementary material 5: NH4-NO3 dynamics over time (PDF 153 kb) [file 248_2024_2456_MOESM5_ESM.pdf]
